# Prevalence of chronic kidney disease and its association with metabolic diseases: a cross-sectional survey in Zhejiang province, Eastern China

**DOI:** 10.1186/1471-2369-15-36

**Published:** 2014-02-21

**Authors:** Bo Lin, Lina Shao, Qun Luo, Lingxia Ou-yang, Fangfang Zhou, Biao Du, Qiang He, Jianyong Wu, Nan Xu, Jianghua Chen

**Affiliations:** 1 Kidney Disease Center, The First Affiliated Hospital, College of Medicine, Zhejiang University, 310003 Hangzhou, P.R.China; 2 Key Laboratory of Nephropathy, Zhejiang Province; Kidney Disease Immunology Laboratory, The Third Grade Laboratory, State Administration of Traditional Chinese Medicine of PR China, 310003 Hangzhou, P.R.China; 3 Division of Nephrology, Ningbo Second Hospital, Ningbo, PR China; 4 Division of Nephrology, Zhoushan Putuo Hospital, Zhoushan, PR China; 5 Division of Nephrology, Quzhou Kecheng Hospital, Quzhou, PR China

**Keywords:** Cross-sectional survey, Chronic kidney disease, Metabolic diseases, Prevalence, Risk factors, Eastern China

## Abstract

**Background:**

The prevalence of chronic kidney disease (CKD) and metabolic diseases has increased at different rates in different regions in China. The aim of our study was to estimate the prevalence of CKD and to analyze associated risk factors of CKD in Zhejiang province, Eastern China.

**Methods:**

A cross-sectional survey of 11,013 adults was conducted from September 2009 to June 2012 in Zhejiang Province, located in Eastern China. CKD was defined as having an estimated glomerular filtration rate (eGFR) < 60 mL/min/1.73 m^2^ or the presence of albuminuria. Medical history, physical examination and laboratory data were used to diagnose metabolic diseases. Age- and sex-standardized prevalence was calculated using the data on the population distribution in China in 2010. We examined risk factors associated with decreased renal function and albuminuria using multivariate logistic regression.

**Results:**

A total of 10,384 adults (94.3%) completed the screening. The standardized prevalence of reduced renal function (eGFR < 60 mL/min/1.73 m^2^) was 1.83% (95% CI 1.52–2.13) and that of albuminuria was 8.65% (95% CI 7.98–9.31). The overall prevalence of CKD was 9.88% (95% CI 9.18–10.59). The prevalence of reduced renal function was greater in the eastern rural areas in Zhejiang province. Multivariate logistic regression revealed that metabolic diseases such as diabetes, obesity, hypertension, and hyperuricemia were independent risk factors of CKD. Patients with metabolic diseases had a significantly (P < 0.001) higher prevalence of CKD than those without such diseases.

**Conclusions:**

CKD has become a severe public health problem in Zhejiang Province, and metabolic diseases may increase the risk of CKD in Zhejiang population.

## Background

The prevalence of chronic kidney disease (CKD) and metabolic diseases such as diabetes, obesity, hyperuricemia, hyperlipidemia and hypertension has increased and has become leading public health problem [1,2]. China is a developing country with more than 30 provinces, autonomous regions and municipalities. Wang et al. reported that the prevalence of chronic kidney disease varied greatly between geographical regions [3]. This might be related to variability in lifestyles and economic development. As a populous and economically developed province in eastern China, the permanent population of Zhejiang reached 54.42 million at the end of 2010. However, there are few reports about the prevalence of CKD in Zhejiang province.

Metabolic diseases such as diabetes, obesity and hypertension are known to be risk factors of kidney injury, and play an important role in the progression of CKD [4-6]. Hypertension and diabetes have been the leading causes of CKD in the developed countries. Approximately 40% of diabetic patients had some degree of CKD in the United States [7] and 40–50% of diabetic patients had kidney injury in Japan [8]. Meanwhile, about 25.3% of patients with hypertension were reported to experience kidney injury in Turkey [9]. Obesity conferred a high susceptibility to CKD in Norway with a relative risk of 1.77 [10].

Metabolic diseases have become major causes of CKD. Many epidemiological studies have found that geographic variation may lead to geographic risk factors for the occurrence of CKD. Geographic attributes may be as diverse as the physical and socioeconomic characteristics of a region and the available medical care [11]. As a big province in a developing country, Zhejiang has its own geographic attributes, but an epidemiological survey studying the association between CKD and metabolic diseases had not been undertaken yet.

Therefore, the purpose of our study was to estimate the prevalence of CKD in Zhejiang province, with a focus on the CKD prevalence in individuals with metabolic diseases, and to analyze the associated CKD risk factors.

## Methods

### Study design and subjects

**Figure 1:**
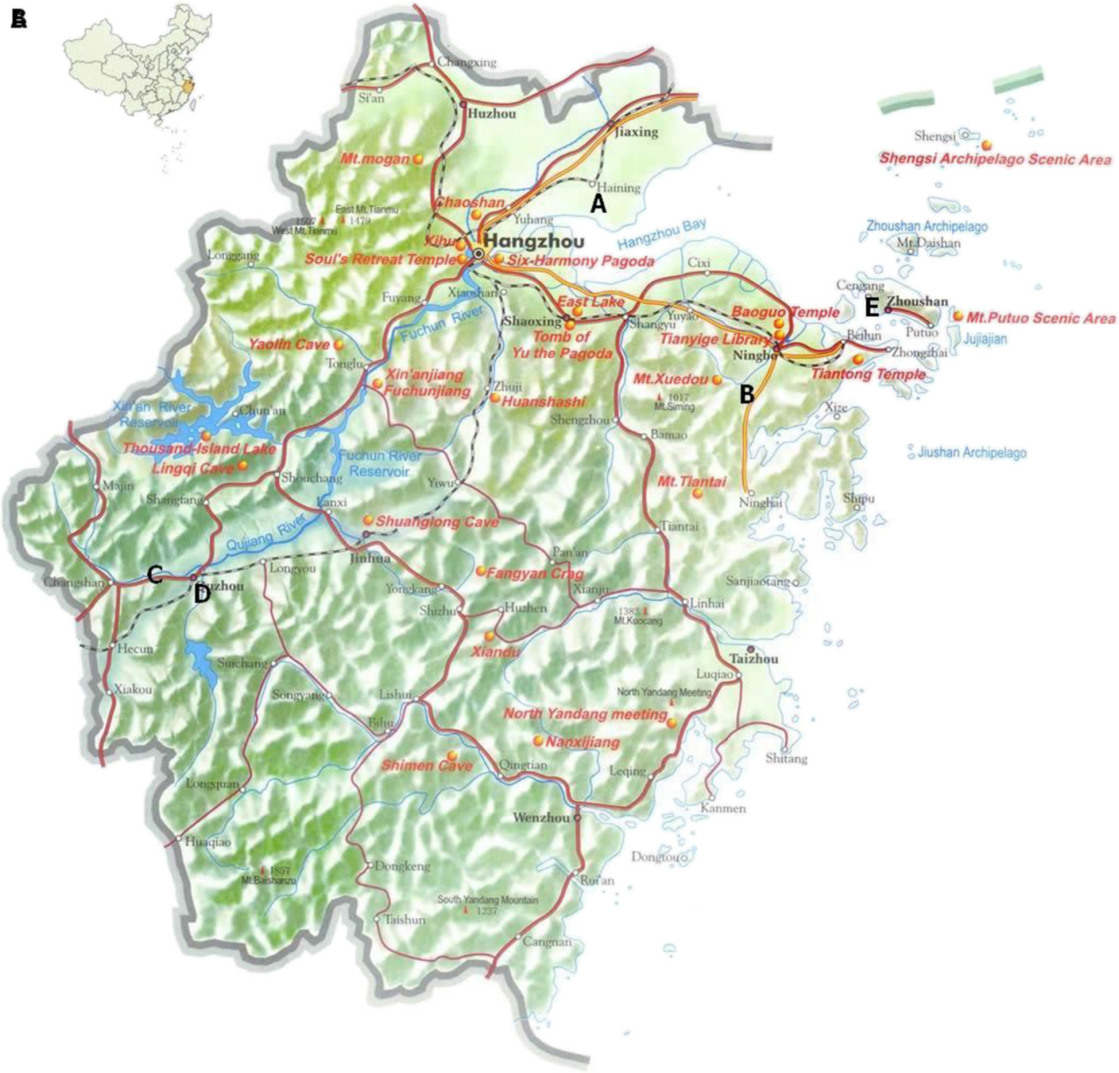
**The Map of Zhejiang Province. A**, **B**, **C**, **D**, and **E** were the five districts we chosen. **A**: eastern rural district. **B**: eastern urban district. **C**: western rural district. **D**: western urban district. **E**: island.

Using a multistage, stratified sampling method, we recruited adult (18+) residents of the Zhejiang province to participate in our cross-sectional survey. First, one urban district and one rural district were randomly selected from the eastern and western regions of the province [12]. Then, from each district, five communities were randomly selected to serve as the population base for this study, for a total of 20 communities. The fishing population of the Zhejiang island was subsequently added to ensure representation of the province’s diverse socioeconomic characteristics [13,14]. Finally, we randomly (by simple randomization using SPSS software, version 19.0) selected 2000–2500 adults from each district (Figure 1) as representative samples. The screening was performed from September 2009 to June 2012. The ethics committee of Zhejiang University First Hospital approved the study. All participants were voluntary and written informed consents were obtained.

### Screening protocol and evaluation criteria

All participants were required to complete a questionnaire including gender, age, family income, diet and medical history, physical examination (height, weight and blood pressure) and laboratory examination (fasting blood glucose, serum creatinine, serum lipids, serum uric acid, urinary albumin and urinary creatinine). All investigators received unified training before screening. Data testing and collection were completed at the local central hospital.

### Chronic kidney disease

Chronic kidney disease was defined as having an estimated glomerular filtration rate (eGFR) of < 60 mL/min/1.73 m^2^ or the presence of albuminuria. Serum creatinine was measured by Jaffe’s kinetic method. The eGFR level was calculated by the simplified Modification of Diet in Renal Disease equation [15]. Urinary albumin and urinary creatinine were measured using a spot morning urine sample. Urinary albumin was measured by the immunoturbidimetric method and urinary creatinine was measured by Jaffe’s kinetic method. The urinary albumin to creatinine ratio (ACR) was calculated. Albuminuria was defined as an ACR value higher than 30 mg/g. Micro-albuminuria was defined as an ACR ranging from 30 to 299 mg/g and macro-albuminuria with an ACR 300 mg/g or greater. CKD was classified based on the levels of eGFR and ACR according to KDIGO criteria [16].

### Blood pressure measurement

Blood pressure was measured by sphygmomanometer at least 3 times with at least a 1 minute interval each time. The mean of these readings was calculated. If the difference between any two readings was greater than 10 mmHg, the mean of the closest two readings was used. Hypertension was defined as a systolic blood pressure (SBP) more than 140 mmHg, a diastolic blood pressure (DBP) more than 90 mmHg, any use of antihypertensive medication in the past 2 weeks irrespective of blood pressure, or a history of hypertension.

### Fasting blood glucose and other measurements

Fasting blood glucose (FBS) was tested with a glucose oxidase method. The diagnosis of diabetes mellitus was established when the FBS was ≥ 7.0 mmol/L, or any use of oral hypoglycemic agents or insulin. Hyperlipidemia was defined as total cholesterol more than 5.18 mmol/L, triglycerides greater than 1.70 mmol/L or pharmacologic lipid-lowering therapy. Hyperuricemia was defined as a serum uric acid level greater than 417 mmol/L in males, or 357 mmol/L in females. Overweight was defined as a BMI of 25.00 to 29.99 kg/m^2^, and obesity was defined as a BMI more than 30.00 kg/m^2^.

### Statistical analysis

Data input and analysis were done by more than two investigators together who had not participated in the screening. Continuous variables were presented as means ± SD, and categorical variables were expressed as proportions with 95% CIs. The standardization prevalence of CKD was adjusted for age and sex based on the population distribution in China in 2010. Differences in demographic and socioeconomic characteristics, and presence of metabolic conditions among participants with and without CKD, were analyzed using two-tailed unpaired student’s t-tests for continuous variables and chi-square tests for categorical variables. We examined risk factors associated with decreased renal function and albuminuria using multivariate logistic regression, and the results were expressed as odds ratios (ORs) with 95% CIs. Age (by decade), sex, hyperuricemia, diabetes, hypertension, obesity, hypertriglyceridemic and hypercholesterolemia as covariates were included in the multivariable logistic regressions. Statistical significance was set at a P value = 0.05. All analyses were done using the SPSS 19.0.

## Results

### Demographic characteristics of the participants

A total of 11,013 people were enrolled in the investigation, with 10,384 people completing the screening for a completion rate of 94.3%. The mean age of all participants was 52.9 ± 14.5 years. The participants with CKD were older than those without CKD (61.0 ± 14.7 years versus 51.6 ±14.0 years, P < 0.001). More women suffered with CKD compared with men (64.5 versus 35.5%). Metabolic characteristics such as FBS, SBP, DBP, total cholesterol (TC), triglycerides (TG), uric acid (UA) and BMI were significantly higher in the CKD group than those in the non-CKD group, all P values < 0.001 (Table 1).

**Table 1:** Clinical features and metabolic characteristics of participants

	**CKD 1**	**CKD 2**	**CKD 3A**	**CKD 3B**	**CKD 4-5**	**CKD 1-5**	**no CKD**	**Total**	P*
**eGFR**	**>90**	**60-89**	**45-59**	**30-44**	**<30**	**<60**	**≥60**		
**ACR**	**≥30**	**≥30**				**or ≥30**	**<30**		
Male n(%)	200(30.1%)	153(35.4%)	101(46.8%)	22(53.7%)	13(56.5%)	489(35.5%)	3974(44.1%)	4463(43.0%)	0.000
Age (years)	58.3 ± 13.5	61.5 ± 14.7	66.5 ± 16.4	66.8 ± 15.1	70.4 ± 10.3	61.0 ± 14.7	51.6 ± 14.0	52.9 ± 14.5	0.000
SBP (mmHg)	146.9 ± 25.7	148.1 ± 25.3	145.2 ± 23.9	149.4 ± 25.8	161.6 ± 28.9	147.3 ± 25.4	131.2 ± 19.9	133.4 ± 21.4	0.000
DBP(mmHg)	84.2 ± 13.4	82.6 ± 12.4	79.5 ± 12.2	80.7 ± 14.2	83.4 ± 9.63	82.8 ± 13.0	78.2 ± 10.8	78.8 ± 11.2	0.000
FBS(mmol/L)	5.69 ± 2.30	5.36 ± 1.76	5.07 ± 1.56	5.41 ± 1.08	4.92 ± 0.87	5.47 ± 2.00	5.03 ± 1.14	5.09 ± 1.30	0.000
TG(mmol/L)	1.70 ± 1.76	1.54 ± 1.09	1.52 ± 1.18	1.83 ± 2.13	1.33 ± 0.71	1.62 ±1.49	1.43 ± 1.60	1.45 ± 1.37	0.000
TC(mmol/L)	5.02 ± 1.08	4.89 ± 0.93	4.97 ± 1.18	4.91 ± 1.08	4.37 ± 0.08	4.96 ± 1.05	4.79 ± 1.42	4.81 ± 1.37	0.000
UA(mmol/L)	275.9 ± 80.4	316.2 ± 92.8	364.4 ± 105.0	393.9 ±113.9	435.9 ± 107.3	308.6 ± 98.1	288.0 ± 86.2	290.7 ± 88.2	0.000
BMI(kg/m2)	23.8 ± 3.85	23.5 ± 4.23	23.2 ± 3.49	23.5 ± 4.22	21.9 ± 3.48	23.6 ± 3.93	23.0 ± 4.00	23.1 ± 3.99	0.000
eGFR(mL/min/1.73m²)	123.7 ± 28.1	73.7 ± 7.91	54.8 ± 3.82	38.5 ± 4.21	16.1 ± 6.62	92.9 ± 37.3	105.8 ± 70.5	104.1 ± 32.7	0.000
ACR(mg/g)	149.7 ± 429.0	196.3 ± 329.6	91.8 ± 259.1	237.2 ± 448.4	354.1 ± 559.8	159.9 ± 380.7	10.0 ± 8.13	29.9 ± 147.8	0.000

CKD was defined as eGFR < 60 mL/min/1.73 m^2^ or albuminuria. Continuous data were presented as means ± SDs and proportions for categorical variables. FBS = fasting blood glucose. BMI = body mass index. SBP = systolic blood pressure. DBP = diastolic blood pressure. TC = total cholesterol. TG = triglycerides. UA = uric acid. SDs = standard deviations. eGFR = estimated glomerular filtration rate. ACR = urinary albumin to creatinine ratio. CKD = chronic kidney disease. Non-CKD: no indicators of kidney damage. P*(comparisons among those with CKD and without CKD).

### Prevalence of CKD

The prevalence was gender- and age-adjusted using data from the population distribution in China in 2010. The adjusted prevalence of albuminuria was 8.65% (95% CI 7.98–9.31) and that of reduced kidney function was 1.83% (95% CI 1.52–2.13). The overall prevalence of CKD was 9.88% (95% CI 9.18–10.59). The prevalence of micro-albuminuria was 7.49% (95% CI 6.88–8.10) and that of macro-albuminuria was 1.16% (95% CI 0.84–1.30). The prevalence of ESRD was 0.06% (95% CI 0.01–0.12). The prevalence of CKD in females was 11.71% (95% CI 10.94–12.48) which was higher than that in males (8.07%, 95% CI 7.73–8.71), P < 0.001 (Table 2).

**Table 2:** Prevalence of chronic kidney disease (CKD) classified by eGFR and albuminuria categories (KDIGO 2012)

	**eGFR (mL/min Per 1.73 m²)**		**A1**		**A2**		**A3**		**Total**
		**n**	**Prevalence (%, 95% CI)**	**n**	**Prevalence (%, 95% CI)**	**n**	**Prevalence (%, 95% CI)**	**n**	**Prevalence (%, 95% CI)**
G1	G1	-	-	605	4.42(3.95-4.89)	59	0.54(0.37-0.70)	664	4.95(4.45-5.45)
G2	G2	-	-	360	2.66(2.30-3.03)	72	0.44(0.29-0.59)	432	3.11(2.71-3.50)
G3	G3	170	1.18(0.93-1.41)	66	0.39(0.24-0.52)	21	0.13(0.05-0.22)	257	1.70(0.40-1.99)
G3a	G3a	151	1.04(0.81-1.27)	52	0.29(0.17-0.31)	13	0.09(0.02-0.16)	216	1.42(1.15-1.69)
G3b	G3b	19	0.14(0.06-0.22)	14	0.10(0.02-0.16)	8	0.04(0.00-0.09)	41	0.28(0.10-0.30)
G4-5	G4-5	10	0.05(0.01-0.12)	4	0.02(0.00-0.05)	9	0.05(0.00-0.12)	23	0.12(0.02-0.24)
Total	Total	180	1.23(0.86-1.48)	1035	7.49(6.88-8.10)	161	1.16(0.93-1.41)	1376	9.88(9.18-10.59)

A1 = Normo-albuminuria: ACR < 30 mg/g; A2 = Micro-albuminuria: ACR 30-300 mg/g; A3 = Macro-albuminuria: ACR > 300 mg/g. The prevalence was adjusted using the data from the population distribution in China in 2010. CKD was defined as eGFR < 60 mL/min/1.73 m^2^ or albuminuria. ACR = albumin-to-creatinine ratio; eGFR = estimated glomerular filtration rate; CKD = chronic kidney disease.

The prevalence of CKD differed between geographical regions in Zhejiang province (Table 3). The prevalence of albuminuria in the eastern urban district was lower than that in the eastern rural district (8.31 versus 8.83%, P < 0.001). However, there was no significant difference on the prevalence of albuminuria between the urban district and rural district in the western Zhejiang province (P = 0.058). Compared with the other areas, the prevalence of reduced renal function in the eastern rural district was the highest (4.83%, all P values < 0.001). The prevalence of reduced renal function in western areas was lower than that in eastern areas (P < 0.001). The residents on the island had a higher prevalence of albuminuria; the prevalence of CKD on the island was the highest in Zhejiang province.

**Table 3:** Prevalence of CKD in difference districts stratified by socio-economic disparities

	**Reduced renal function**	**Albuminuria**	**CKD**
	**(95% CI)**	**(95% CI)**	**(95% CI)**
Eastern			
Rural residents (A)	4.83(4.34-5.33)	8.83(8.16-9.50)	12.20(11.41-12.98)
Urban residents (B)	1.86(1.55-2.17)	8.31(7.66-8.96)	9.49(8.79-10.18)
Western			
Rural residents (C)	0.53(0.36-0.69)	8.46(7.81-9.16)	8.69(8.03-9.36)
Urban residents (D)	0.42(0.28-0.57)	9.15(8.49-9.83)	9.32(8.63-10.01)
Island residents (E)	1.88(1.57-2.19)	11.72(10.95-12.49)	12.95(12.12-13.76)

Prevalence was gender and age adjusted. Eastern: the eastern part of Zhejiang province. Western: thewestern part of Zhejiang province .Albuminuria was defined as an ACR >30 mg/g. CKD wasdefined as eGFR < 60 mL/min/1.73 m^2^ or albuminuria. Reduced renal function = eGFR < 60 mL/min/1.73 m^2^.

### Associated risk factors for CKD

Multivariate analysis by binomial logistic regression demonstrated that the following factors were independently associated with the presence of albuminuria: age (by decade), female, hypertension, diabetes, hyperuricemia and obesity (Table 4). Hypercholesterolemia was not an independent risk factor of micro-albuminuria (OR = 0.93, 95% CI 0.80–1.08, P = 0.317). Only age (by decade), hyperuricemia and albuminuria were significantly associated with reduced renal function, all P values < 0.001.

**Table 4:** Multivariate logistic regression analysis of factors associated with chronic kidney disease (CKD)

	**Micro-albuminuria**		**Macro-albuminuria**		**Albuminuria**		**G3- G5**	
	**OR (95% CI)**	**P**	**OR (95% CI)**	**P**	**OR (95% CI)**	**P**	**OR (95% CI)**	**P**
Age (change by 10 years)	1.36(1.29-1.45)	<0.001	1.38(1.20-1.59)	<0.001	1.91(1.29-1.44)	<0.001	1.90(1.70-2.14)	<0.001
Femal	1.79(1.56-2.07)	<0.001	1.87(1.33-2.63)	<0.001	1.81(1.58-2.06)	<0.001	0.98(0.76-1.27)	0.883
Hypertension	2.45(2.11-2.85)	<0.001	2.35(1.63-3.40)	<0.001	2.44(2.12-2.82)	<0.001	1.03(0.78-1.36)	0.820
Diabetes	1.88(1.47-2.41)	<0.001	1.94(1.12-3.34)	0.017	1.89(1.49-2.39)	<0.001	0.54(0.29-1.02)	0.059
Obesity	1.36(1.10-1.69)	0.005	1.59(1.01-2.51)	0.047	1.39(1.14-1.70)	<0.001	0.86(0.55-1.33)	0.490
Hyperuricemia	1.32(1.08-1.62)	0.007	2.22(1.48-3.34)	<0.001	1.43(1.19-1.73)	<0.001	5.66(4.32-7.42)	<0.001
Hypertriglyceridemic	1.06(0.91-1.23)	0.492	0.90(0.62-1.29)	0.556	1.03(0.89-1.20)	0.671	0.89(0.66-1.18)	0.410
Hypercholesterolemia	0.93(0.80-1.08)	0.317	1.39(1.00-1.94)	0.049	0.99(0.86-1.13)	0.839	0.90(0.69-1.18)	0.447
Albuminuria	-	-	-	-	-	-	2.82(2.13-3.73)	<0.001

Albuminuria: ACR > =30 mg/g. Micro-albuminuria: ACR30-299 mg/g. Macro-albuminuria: ACR 300 mg/g or more; G3- G5 = eGFR <60 mL/min/1 · 73 m^2^. OR = odds ratios.

**Figure 2:**
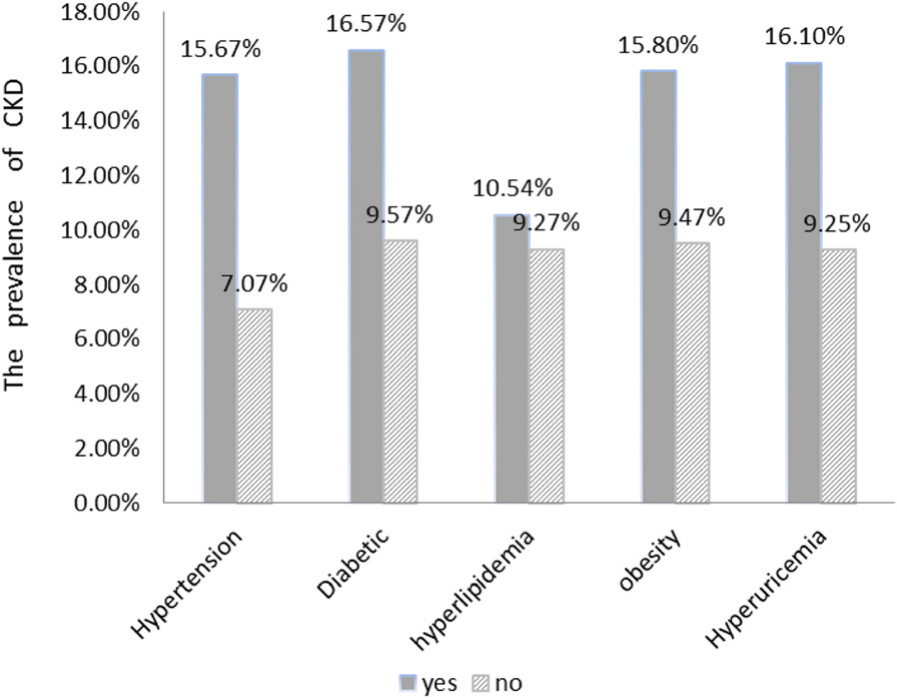
**The prevalence of CKD in subjects with different metabolic diseases.** Data were adjusted prevalence. yes = subjects with the disease. no = subjects without the disease.

The prevalence of CKD in patients with hypertension was higher than that in individuals without hypertension (P < 0.001). Likewise, there were significant differences in the prevalence of CKD between people with diabetes and non- diabetics (P < 0.001), obesity and non-obesity (P < 0.001), hyperuricemia and non-hyperuricemia (P < 0.001). The comparison between patients with hyperlipidemia and those without hyperlipidemia showed there was significant difference in the prevalence of CKD with presence of hyperlipidemia (10.54 vs 9.27%, respectively, P < 0.001) (Figure 2).

## Discussion

### Prevalence of CKD

To the best of our knowledge, this study was the first epidemiological survey of CKD in Zhejiang province. The overall prevalence of CKD in Zhejiang province (9.88%) was lower than results from a national survey (10.8%). Several cross-sectional studies indicated that the CKD prevalence differed substantially between geographical regions in China [17-19]. The prevalence of CKD in Beijing was 13.0%, which was much higher than the results we found for Zhejiang province. In the study from Zhuhai, Southern China, the overall prevalence of CKD was 12.5%. In Shanghai, the prevalence of CKD was 11.8%, which was also higher than our present study. Besides the differences in study methodology, this heterogeneity might be related to variability in socioeconomic characteristics. China is a developing country with 34 provinces, autonomous regions and municipalities. Significant differences in lifestyles, climatic conditions, medical care and economic development between geographical regions may cause the differences in the diseases’ prevalence. Xu et al. [20] reported that the prevalence of diabetes in Chinese adults increased with economic development. Wang et al. reported that the prevalence of CKD varied greatly between geographical regions.

In the analysis of different regions in Zhejiang, the eastern rural region had a rather high prevalence (4.83%) of reduced kidney function. In our study, eGFR was calculated using serum creatinine. To confirm these results, serum creatinine was measured again in the central laboratory of Zhejiang University First Hospital, and with the same results. The differences of socioeconomic status (lifestyles, medical insurance and family income) between the eastern and western regions of Zhejiang may have led to the variation in CKD [12], but these factors were not fully captured in our survey and deserve further investigation. Additionally, the high prevalence of hyperuricemia in this region may contribute to the high prevalence of reduced renal function. We found that the prevalence of hyperuricemia in the eastern rural region was greater than that in other regions. Hyperuricaemia was reported to increase the risk for new-onset kidney disease [21,22]. The island residents had the highest prevalence of CKD in Zhejiang province. This may be because of the high sodium diet and unhealthy lifestyles, as a survey of lifestyles reported diet on the island of Zhejiang is typically high in sodium [14]. High sodium intake is associated with increased micro-albuminuria and decreased renal function [23].

### The risk factors of CKD

Our study suggests that metabolic diseases such as diabetes, obesity, hypertension, and hyperuricemia were independent risk factors of CKD. These results are consistent with previous studies [24,25]. Zhejiang has undergone rapid social-economic development in the past decades. A rapid increase in metabolic diseases has taken place, which may be related to the changed lifestyles. Nationwide surveys of hypertension suggested that the prevalence of hypertension increased from 11.3 to 26.6% in the Chinese adult population ([26,27], and the prevalence of diabetes increased from 5.5 to 9.7% [28,29]. It suggests that diabetes has been the most common underlying cause for CKD [30]. Obesity prevalence has doubled over the last 3 decades [31], and the rapid increase in the prevalence of metabolic diseases may result in an even greater burden of CKD. In our survey, patients with hypertension, diabetes, obesity and hyperuricemia had a higher prevalence of CKD than those without these diseases. For example, the prevalence of CKD in diabetics was 15.7%, which was only 7.07% in non-diabetics. Thus, slowing the rise in metabolic diseases may be a way of preventing CKD. Nevertheless, more studies are needed to confirm this hypothesis.

### Limitations

Our study has certain limitations and constraints. We did not measure HDL and LDL in our survey. High LDL cholesterol may be an independent risk factor of CKD. Our study also relied on single measurements for eGFR and albuminuria without repeated examination, because it was cumbersome and expensive, and that may have resulted in misclassification of individuals with CKD. Additionally, the cross-sectional design of the study makes causal inferences impossible.

## Conclusions

CKD is an important public health problem in Zhejiang province, and metabolic diseases may increase the risk of CKD in Zhejiang population.

## Competing interests

The authors declare that they have no competing interests.

## Authors’ contributions

WJY and CJH conceived of the study, and participated in its design and coordination and helped to draft the manuscript. ZFF, LQ, OYLX, and DB collected the data. LB and SLN participated in the design of the study, analyzed the data, interpreted the results, and wrote the manuscript. HQ and XN revised the report. All authors read and approved the final manuscript.

## Pre-publication history

The pre-publication history for this paper can be accessed here:

http://www.biomedcentral.com/1471-2369/15/36/prepub
